# Editorial: Re-emergence of natural products for drug discovery in honor of Prof. Dr. M. Iqbal Choudhary

**DOI:** 10.3389/fphar.2023.1227732

**Published:** 2023-07-03

**Authors:** Hidayat Hussain, Hina Siddiqui, Ioannis P. Gerothanassis

**Affiliations:** ^1^ Department of Bioorganic Chemistry, Leibniz Institute of Plant Biochemistry, Halle, Germany; ^2^ H.E.J. Research Institute of Chemistry, International Center for Chemical and Biological Sciences, University of Karachi, Karachi, Pakistan; ^3^ Department of Chemistry, Section of Organic Chemistry and Biochemistry, University of Ioannina, Ioannina, Greece

**Keywords:** editorial, natural product, drug discovery, plants, cancer

Nature has always been a notable source of bioactive lead compounds and has provided unprecedented opportunities for medicinal chemists to continuously provide drug candidates as new compounds for evaluation. Natural products (NPs) and their derivatives have historically made major contributions to pharmacotherapy, in particular for cancer and infectious diseases. NPs provide intriguing chemical diversity and are considered as being irreplaceable sources of inspiration for new drug discovery and have been validated by their truly enormous contributions in the development of new lead molecules. These secondary metabolites feature diverse chemical structures, and inherently occupy biologically applicable chemical spaces. Notably, NPs and their analogs are responsible for over half of all approved drugs in current use. FDA data illustrated that NPs and their analogs represent more than one-third of all FDA-approved new lead molecules (Li and Lou, 2018).

Many renowned scientists have contributed to this topic, which includes 13 papers, original articles along with review articles that give the readers of the Frontier in Pharmacology an updated and new appreciation of the tremendous role played by natural products in drug discovery.

Hypericin is a penanthroperylenequinone which is a naturally occurring secondary metabolite produced by some *Hypericum* species. Hypericin was initially produced by *H. perforatum* (Brockmann et al., 1939), generally called St. John’s wort, which is one of the most important members of the *Hypericum* genus. Numerous cancers including myeloid leukemia, prostate cancer, glioblastoma, and breast cancer demonstrate powerful chemo resistance which is assisted by increased expression of numerous anti-apoptotic Bcl-2 proteins. Therefore one of the more important anti-cancer strategies would be to develop inhibitors of these Bcl-2 proteins, the BH3 mimetics. Stroffekova and coworkers (Doroshenko et al.) have demonstrated that hypericin can cause critical activity in the mitochondria function and cellular ultrastructure, and also cause distribution of Bcl2 proteins. The authors proposed that the possible mechanisms of cytotoxic effects could be due to the direct interconnection between hypericin and Bcl-2 proteins. Computational chemistry confirmed this hypothesis that hypericin interacts with BH3 and BH1 peptides.

The plant *Origanum syriacum* is employed to treat respiratory and gastrointestinal diseases in Syrian folk medicine. In addition, this plant is used to treat various respiratory diseases in Palestine, Israel and Jordan. Moreover *O. syriacum* is employed in Jordan as a pectoral, carminative, aperitif, antitussive and as an anti-stomachache. Additionally *Origanum* species were used to treat hemorrhoids, sexual diseases, internal diseases, animals bites, pains and poison in various Arab countries (Lukas et al., 2009). Mesmar et al. investigated the phytochemical content and anticancer effects of an ethanolic extract of *O. syriacum*. The authors demonstrated that this extract showed promising antioxidant effects and induced the generation of ROS in breast cancer cells (MDA-MB-231 cells). Mechanistic studies demonstrated that an *O. syriacum* extract induced G0/G1 cell cycle arrest, associated with p38 MAPK phosphorylation, protein p21enhancement and reduction of Ki67 protein. Moreover, the extract decreased the invasive and migration potentials of MDA-MB-231 cells via the deactivation of FAK (focal adhesion kinase).

*Brassica rapa* belongs to the family *Cru-ciferae*. The literature about this plant revealed that it demonstrated antimicrobial, antioxidant, antitumor, antiinflammatory, cardioprotective, hypolipidemic hepatoprotective, antidiabetic, nephroprotective, and analgesic effects (Paul et al., 2019). Abid et al. evaluated the antihypertensive effects of a methanolic extract the leaves of *B. rapa* and found that the highest concentration of natural products including polyphenols, flavonoids and polysaccharides to be present while an aqueous extract was rich in saponins. Moreover, the aqueous extract demonstrated promising antihypertensive potential by illustrating inhibition towards the angiotensin-converting enzyme (ACE). GC-MS analysis revealed that oleic acid was present in the *B. rapa* methanolic extract and docking analysis confirmed that this molecule is the main constituent responsible for antihypertensive effects.

*Polygonum multiflorum* is employed in Chinese traditional medicine to treat leptotrichia, hyperlipidaemia, inflammation and learning and memory obstructions. This plant was also reported to show hair-blacking and liver-tonic effects. Furthermore, this plant is employed in traditional medicine to treat sore scabies, pruritus, ringworm, carbuncles, scrofula, and postpartum (Lin et al., 2015). 2,3,5,4′-Tetrahydroxystilbene-2-O-β-D-glucoside (THSG) is a stilbene glycoside reported from *P. multiflorum* and literature revealed that THSG illustrated various biological effects, such as hepatoprotection, cardiovascular protection, memory enhancement, neuroprotection, anti-aging, anti-cancer, anti-oxidation, anti-osteoporosis, and anti-inflammation (Huang et al.). Chong and coworkers (Huang et al.) investigated the protective effect of THSG towards bleomycin-induced lung fibrosis. The authors found that THSG potentially attenuated lung injury via decreasing fibrosis and extracellular matrix deposition. Further studies revealed the promise that this stilbene downregulated fibronectin, TGF-β1, CTGF, α-SMA, and TGFBR2. On the other hand, THSG upregulated SOD-1, LC3B, and catalase in the lungs of treated mice. Mechanistic studies demonstrated that THSG potentially decreased TGF-β1-induced enhancement of TGFBR2 expression and phosphorylation of Akt, Smad2/3, ERK1/2, and mTOR in MRC-5 cells.

The bacteria species *Streptomyces* produced numerous anti-infective potential NPs. These bacteria provided natural products with significant medical efficacy. For instance, ivermectin isolated from S. avermitilis is largely employed for the treatment of helminth infections. In addition, paromomycin, aminoglycoside, isolated from S. rimosus is used for the treatment of various parasitic infections including leishmaniasis. Awada et al. evaluated the anti-leishmanial effects of various extracts of *Streptomyces sp*. isolated and showed potent anti-leishmanial effects towards L. tropica. Based on bio-guided fractionation the isolation and structure elucidation of a natural product named “HAS1” which possess an acetogenins skeleton was isolated. Furthermore the isolated compound showed promising anti-leishmanial effects towards *L. tropica*.

Al-Hassan et al. phytochemically investigated the edible catfish Arius bilineatus and isolated twelve oxysterols including two deoxygenated steroids and cholesterol. Moreover these steroids were screened for their cytotoxic effects towards three human cancer cells including the CML cell line (K-562), breast cancer cells (MDA MB-231 and MCF-7). Cholesta-3,5,6-triol was the most active steroid towards K562 leukemic cells. Notably, steroids which featured the 5,6-epoxy group were inactive, indicating that the epoxide acquire its cytotoxic effects only after being hydrolysed to the corresponding diol precursor. Furthermore SAR showed that the importance of the C-3 hydroxyl group along with either a C-7 OH moiety and the unsaturation at C-5/C-6 or the C-5/C-6 OH groups might be replaced by a unsaturation linked with a C-7 OH group.

Filimonova and coworkers identified T1059, 1-cyclohexanoyl-2-ethylisothiourea hydrobromide and illustrated its potent eNOS and iNOS inhibitor activity along with its vasotropic effects. In an initial investigation in rats, T1059 demonstrated significant vasoconstrictive effects at relatively safe doses but was accompanied with an increase in peripheral vascular resistance in rats (Filimonova et al., 2018). In another study the same authors demonstrated that T1059 showed long-term vasoconstrictor effects (Filimonova et al., 2020) and that this molecules did not cause a potential baroreflex response in rats. Quite recently Filimonova and coworkers (Filimonova et al.) screened the T1059 vasopressor effects in rats and dogs and demonstrated significant vasoconstrictor effects. Treatment of T1059 in dogs and rats induced and enhancement in vascular tone along with significant hypertensive effects. Moreover the low dose administration of T1059, inhibited the establishment of cardiorespiratory disorders and potentially increased the survival of animals.

The COVID-19 pandemic has resulted in 639 132 486 cases being reported in addition to 6 614 082 deaths around the globe during the last 3 years. (World Health Organization, 2022). The various variants including Alpha, Beta, Gamma, Delta, and Omicron of the SARS-CoV-2 are thought to be responsible for all these cases and deaths. Natural products continuously play a tremendous role in drug discovery including for COVID-19 and are still considered as a most important strategy to defeat this ongoing pandemic. Wang et al. published a review article about the role of natural products in COVID-19 therapy. The authors included various natural products with intriguing effects towards SARS-CoV-2 and related viruses. They discussed the effects of various natural products as anti-COVID-19 treatment from numerous natural product classes including alkaloids, peptides, steroids, triterpene glycosides, xanthones, diterpenes, macrolides, cannabinoids, flavones, coumarins, and naphthoquinones.

Zhou et al. published an interesting review on the biological and pharmacological effects of sclareol. They described how sclareol, a diterpene reported from *S. sclarea* is applied as a traditional medicine to treat oral inflammation, arthritis, dysmenorrhea, and digestive system diseases. Moreover sclareol illustrated significant anticancer effects towards various cancer cells including leukemia (HL60), breast (MN1, MDD2, MCF-7), colon (HCT116), cervical (HeLa), lung (H1688, A549), and osteosarcoma (MG63). Additionally, sclareol illustrated anti-pathogenic and anti-inflammation effects.

Liu et al. published a comprehensive review on significant drug targets and treatments which affect oxidative stress in gliomas diseases. The authors discussed oxidative stress and its link with gliomas along with pathogenesis of gliomas and the perceived mode of action of oxidative stress in gliomas. In addition, the authors described various natural products that can be used to treat gliomas. These natural products include thymoquinone, chidamide, atovaquone, ivermectin, chloroquine, quinacrine, melatonin, chaetocin, celastrol, shikonin, chaetocin and cannabidiol. Guerra et al. described in their review article that cannabinoids have significant effects on the audiovestibular function. The authors demonstrated that the cannabinoid receptors expression in the audiovestibular pathway demonstrates their tremendous role in drug development. Notably, these drugs may be fruitful for diseases including ototoxicity, noise-induced hearing loss, and various forms of vertigo.

Gaobotse et al. provide an overview some African medicinal plants which demonstrated anticancer properties. These plants include *Dicoma anomala, Portulaca oleracea, Withania somnifera, Azanza garckeana, Cajanus cajan, Combretum caffrum, Prunus avium, Prunus Africana, Securidaca longipedunculata, Annona muricata, Annona senegalensis, Aerva javanica, Abelmoschus esculentus, Flueggea virosa, Lagenaria siceraria, Xylopia aethiopica, Nymphaea lotus, Zanthoxylum chalybeum, Ceratonia siliqua, Moringa oleifera, and Peganum harmala*. In another article, Badivi et al. developed the formulation of bee venom-loaded liposomes coated with PEG (BV-Lipo-PEG). Cytotoxic reults demonstrated that bee venom has ablity to enhance the cytotoxic effects towards various cancer cells. Additionally, BV-Lipo-PEG reduce the expression levels of cyclin E genes, MMP-9, and MMP-2 while enhanced the expression level of caspases 3 and 9.
